# Genome-Wide Identification, Characterization and Expression Profiling of Potato (*Solanum tuberosum*) Frataxin (*FH*) Gene

**DOI:** 10.3390/genes14020468

**Published:** 2023-02-11

**Authors:** Firat Kurt, Ertugrul Filiz, Kubra Yildiz, M. Aydın Akbudak

**Affiliations:** 1 Department of Plant Production and Technologies, Faculty of Applied Sciences, Mus Alparslan University, 49250 Mus, Turkey; 2 Department of Crop and Animal Production, Cilimli Vocational School, Duzce University, Cilimli, 81750 Duzce, Turkey; 3 Department of Agricultural Biotechnology, Akdeniz University, 07058 Antalya, Turkey

**Keywords:** frataxin, mitochondria, iron homeostasis, sulfur, transcription factors, co-expression

## Abstract

Frataxin (FH) plays a crucial role in the biogenesis of mitochondria and the regulation of iron in the cells of various organisms. However, there has been very little research on FH in plants. In this study, the potato *FH* gene (*StFH*) was identified and characterized using a genome-wide approach, and its sequence was compared to those of *FH* genes from *Arabidopsis*, rice, and maize. The *FH* genes were found to have a lineage-specific distribution and were more conserved in monocots than in dicots. While multiple copies of *FH* genes have been reported in some species, including plants, only one isoform of *FH* was found in potato. The expression of *StFH* in leaves and roots was analyzed under two different abiotic stress conditions, and the results showed that *StFH* was upregulated more in leaves and that its expression levels increased with the severity of the stress. This is the first study to examine the expression of an *FH* gene under abiotic stress conditions.

## 1. Introduction

Iron represents a vital nutrient for all living organisms. In fact, it functions as a cofactor in several critical cellular processes, including oxygen transport, photosynthesis, respiration, DNA replication, DNA repair, and amino acid synthesis [1]. Iron deprivation has been shown to dramatically impair these pathways, whereas iron overload has been found to result in oxidative stress as well as irreversible cell and tissue damage [1,2,3]. Therefore, iron homeostasis is essential for ensuring both optimum development and a healthy lifespan.

Frataxin (FH) is a ubiquitous mitochondrial protein known to prevent the oxidative stress that results from excess iron accumulation and iron detoxification [4,5,6]. It is very highly conserved in both prokaryotes and eukaryotes [7,8]. Moreover, FH plays an important role in the oversight of Fe storage, Fe–S cluster assembly, heme metabolism, respiration, and reactive oxygen species (ROS) regulation. FH deficiency can result in several metabolic problems, sensitivity to oxidative stress, and a decreased lifespan [9,10]. A recent study revealed that FH also serves to prevent biotic stresses in plants [11]. More specifically, having transformed to overexpress the *AtFH* gene, *Arabidopsis* (*Arabidopsis thaliana*) plants were found to have developed basal resistance and become resistant against *Pseudomonas syringae* pv. tomato DC3000, which indicated the role of FH in plant immunity [11].

To date, the research concerning FH in plants has been very limited. One *FH* gene in *Arabidopsis* [12] and two homologous *FH* genes in maize (*Zea mays*) (*ZmFH1* and *ZmFH2*) have been identified genome-wide [4]. The *Arabidopsis FH* (*AtFH*) was the first *FH* gene to be identified in plants, sharing 65% homology with the human *FH* gene. The protein encoded by the *AtFH* gene contains a transit peptide for mitochondrial localization [6]. As mentioned previously, FH deficiency leads to an increase in the free-iron content within the mitochondria, thereby causing an increase in both ROS formation and ROS-detoxifying enzymes, such as superoxide dismutase (SOD) and nitric oxide synthase (NOS) [1,5,13]. The *Arabidopsis* plants in which the *FH* gene was knocked out showed lethal embryo phenotypes, which indicated FH to be essential in seed formation. The FH knockdown in *Arabidopsis* resulted in a reduction in *FH* transcription and translation. In the knockdown mutant, the mitochondrial enzymes containing Fe–S, for example, mitochondrial aconitase and succinate dehydrogenase, were reduced [11]. A decrease in the FH level in this mutant resulted in elevated iron accumulation. In addition, recent studies have revealed that FH plays a role not only in the mitochondria but also in the chloroplasts, with FH deficiency altering the normal functioning of the chloroplasts [4,14].

The yield of potato (*Solanum tuberosum*) is severely affected by drought and salt stresses [15,16,17]. There is a positive correlation between the length of potato leaves and the water potentials of the leaf [18]. During drought stress, the leaves grow less, and their surface areas are smaller, reducing the photosynthesis rate. Tuber size, in return, is highly affected by water shortage following tuber initiation [19]. Salt stress, on the other hand, reduces water movement and causes nutrient imbalances in potato [20]. It causes a decrease not only in yield but also in the quality of tubers [21,22,23]. The yield loss may reach 60%, owing to the inhibition of tuberization under salinity stress [24].

Abscisic acid (ABA) plays a crucial role in regulating plant response to drought stress [25,26]. It is involved in the opening and closing of stomata and acts as an endogenous messenger in the regulation of the plant’s water state [27,28]. ABA is considered a plant stress hormone, as various environmental stresses trigger its synthesis [29,30]. In addition, ABA is transported to the leaves and causes the closure of stomata, inhibiting plant growth and helping the plant adapt to adverse conditions [31]. Transcription factors are key regulators in signal transduction and gene expression regulation under environmental stress conditions [32,33]. ABA-inducible genes contain conserved cis-acting elements, called abscisic acid-responsive elements (ABREs), in their promoter region [34,35,36]. Several transcription factors, such as *DREB2A/2B*, *AREB1*, *RD22BP1*, and *MYC/MYB*, are known to regulate ABA-responsive gene expression by interacting with *cis*-acting elements, such as DRE/CRT, ABRE, and MYCRS/MYBRS, respectively [35,37,38,39]. Other transcription factors, such as *AtGT2L* and *ZFHD1*, have also been studied for their role in responding to cold and salt stress and osmotic stress, respectively [40,41,42].

In the present study, we identified a single FH gene (*StFH*) on the seventh chromosome of the potato genome. Moreover, we conducted diverse bioinformatics analyses to achieve its characterization. Containing around 27,000 genes expressed under abiotic, biotic, and hormonal treatments, the co-expression networks of *StFH* were constructed at a coefficient level greater than 0.99 (*r* > 0.99). The transcription factors (TFs) that induced *StFH* expression were also identified in the above-mentioned networks in order to determine the probable candidate genes involved in the same or similar pathways to the *StFH*. The comparative bioinformatics analyses revealed the differences and similarities among the *Arabidopsis* (*Arabidopsis thaliana*), rice (*Oryza sativa*), and maize (*Zea mays*) *FH* genes/proteins. This study is one of the few to investigate the role of frataxin (FH) genes in plants, and it is the first to examine the expression of an FH gene under two major abiotic stresses: drought and salt stresses.

## 2. Materials and Methods

### 2.1. Sequence Identification, Collection, and Analyses

The reviewed sequence of *Arabidopsis* frataxin (FH) peptide (Q9ZR07) from the UniProtKB/Swiss-Prot database (https://www.uniprot.org/, accessed on 1 May 2021) was used to find FH homologs in *Solanum tuberosum* (potato, St), *Oryza sativa* (rice, Os), and *Zea mays* (maize, Zm) genomes via a BLASTp search in Phytozome v12.1 (https://phytozome.jgi.doe.gov/pz/portal.html, accessed on 1 May 2021) [43]. The FHCyay domain (PF01491) of the proteins was validated on the Pfam 35.0 database [44] (http://pfam.xfam.org/, accessed on 1 May 2021). The domain structure found was validated using the conserved domains server (https://www.ncbi.nlm.nih.gov/Structure/cdd/wrpsb.cgi, accessed on 1 May 2021) [45]. Moreover, the genomic, coding, and amino acid sequence data of *FH* genes were retrieved for sequential and structural analyses of the proteins. The coding sequences were analyzed using DnaSPv6 [46], including GC content, numbers of polymorphic (segregating) sites, parsimony-informative sites, nucleotide diversity, and Tajima’s D statistics [47]. The physiochemical properties of FH proteins were identified on the ProtParam server (http://web.expasy.org/protparam) [48]. The sub-cellular localizations of FH proteins were predicted on the WoLFPSORT server [49] (https://wolfpsort.hgc.jp/, accessed on 1 May 2021). The BioEdit v7.2.5 program was employed to reveal the sequence identity matrices and the amino acid compositions of FH proteins [50]. The NetPhos 3.1 server (http://www.cbs.dtu.dk/services/NetPhos/, accessed on 1 May 2021) was used to predict phosphorylation sites in the FH proteins [51].

### 2.2. Phylogenetic and Gene Ontology Analyses

The phylogenetic tree was drawn using the neighbor-joining (NJ) method [52]. The evolutionary distances in the tree were assumed using the Poisson correction method. The phylogenetic analyses were performed in MEGA7 [53]. The functional annotation and gene ontology (GO) classes of StFH protein were processed using the PANNZER (Protein ANNotation with Z-scoRE) server (http://ekhidna2.biocenter.helsinki.fi/sanspanz/, accessed on 1 May 2021) [54].

### 2.3. Predicted Cis-Elements

The predicted transcription factor binding sites (TFBSs) in the 2000-bp upstream region of the promotor were checked using the plant promoter analysis navigator (PlantPANv3; http://PlantPAN.itps.ncku.edu.tw, accessed on 1 May 2021) [55]. Additionally, the PLACE database (https://www.dna.affrc.go.jp/PLACE/, accessed on 1 May 2021) was used to identify cis-acting regulatory DNA elements related to various stress responses [56]. 

### 2.4. In Silico Gene Expression and Synteny Analyses

The digital gene expression data were obtained from the RNA-Seq database of the Potato Genome Sequencing Consortium [57]. The data comprise three different expression datasets, each of which includes 39,031 genes. The first dataset includes gene expressions under *Phytophthora infestans* infection and some elicitors, including acibenzolar-S-methyl (BTH) and DL-b-amino-n-butyric acid (BABA) (hereafter biotic treatment or dataset). The second dataset includes gene expressions under the following abiotic treatments: salinity (150 mM NaCl), drought (mannitol 260 mM), and heat (35 °C) (hereafter abiotic treatment or dataset). Lastly, the third dataset is composed of expression data under hormone treatments (hereafter hormone dataset), such as abscisic acid (ABA; 50 µM), 6-benzylaminopurine (BAP; 10 µM), gibberellic acid (GA3; 50 µM), and indole-3-acetic acid (IAA; 10 µM). The RNA-Seq data analysis was conducted on the Galaxy platform [58] according to the Tuxedo protocol [59]. Concisely, a quality check was applied to the raw data, and the data were trimmed. The trimmed data were again quality-checked by TopHat, and Bowtie was used for the alignment against the potato genome. Cufflinks were used for transcript assembly, and Cuffdiff was used to calculate gene expressions.

### 2.5. Protein Interaction Network, Secondary and Tertiary Structure Analyses

The putative protein-protein interaction (PPI) network was drawn using the STRING v11 server (https://string-db.org/, accessed on 1 May 2021) [60]. The secondary structure analyses of FH proteins were conducted on the SOPMA server (https://npsa-prabi.ibcp.fr/cgi-bin/npsa_automat.pl?page=/NPSA/npsa_sopma.html, accessed on 1 May 2021) [61]. The predicted three-dimensional (3D) structures were generated by the Phyre^2^ server (http://www.sbg.bio.ic.ac.uk/phyre2/html/page.cgi?id=index, accessed on 1 May 2021) [62]. The structure validations were checked using the Ramachandran plot [63] (http://vadar.wishartlab.com/, accessed on 1 May 2021) and the QMEAN Z-score analyses [64] (https://swissmodel.expasy.org/qmean/, accessed on 1 May 2021). The structural overlaps of FT proteins were calculated by pairwise alignment using the CLICK server (http://cospi.iiserpune.ac.in/click/, accessed on 1 May 2021) [65]. The binding site of FT proteins was predicted on the InterProv75 server (http://www.ebi.ac.uk/interpro/, accessed on 1 May 2021). The numbers of the channels were estimated by the BetaCavityWeb server [66] (http://voronoi.hanyang.ac.kr/betacavityweb/, accessed on 1 May 2021).

### 2.6. Plant Materials and Stress Treatments

The potato (*S. tuberosum*) cultivar ‘Agria’ was used in this study. The plantlets were sub-cultured on MS-0 media (4.4 g MS salts, 3% sucrose, 0.7% agar, pH 5.7) and maintained in a growth chamber under 16 h light / 8 h dark photoperiod at 22 °C and 70% relative humidity. Five weeks later, the plantlets were transferred to the hydroponic systems in a growth chamber containing Hoagland’s solution (pH 5.8). Following 2 days of acclimatization, the solutions in the hydroponic systems were replaced with fresh Hoagland’s solution containing 25% PEG-6000 or 200 mM NaCl for the drought stress or salt stress treatments, respectively. The Hoagland solution of the control plants was also refreshed simultaneously. After 24 h of the drought stress and salt stress treatments, the plant leaves and roots were harvested for RNA isolation and physiological assays.

### 2.7. Assaying Proline and MDA (Malondialdehyde) Contents

To assay the proline content of the tissues, 0.5 g of leaf or root tissue was grounded in liquid nitrogen and suspended in 10 mL of 3% (*v/v*) sulfosalicylic acid. The suspensions were filtered through filter papers (Whatman, Florham Park, NJ, USA). Two ml of each filtrate were mixed with 2 mL acid ninhydrin and 2 mL glacial acetic acid and kept at 95 °C for one hour. The reaction was terminated by placing the mixes on ice, and 4 mL of toluene was added to each. The chromophore phases were transferred into quartz cuvettes, and the absorbances were measured at 520 nm using a spectrophotometer [67].

The MDA content of the plants was assayed using a method modified from that of Ohkawa et al. [68]. Grounded in liquid nitrogen, 0.2 g of leaf or root tissue was suspended in 2 mL of 5% trichloroacetic acid (TCA). The homogenates were transferred into clean 2 mL microfuge tubes and centrifuged at 12,000 rpm at room temperature. Equal amounts of lysate and freshly prepared 0.5% thiobarbituric asit (TBA) in 20% TCA were mixed and incubated at 96 °C for 25 min. The tubes were chilled on ice until they reached room temperature and centrifuged at 10,000 rpm for 5 min. The absorbances of the supernatants were measured at 532 nm and 600 nm wavelengths to clear off non-specific reflections due to turbidity. A freshly prepared 0.5% TBA in 20% TCA solution was used as blank. The MDA content of the samples was assayed using an absorbance coefficient of 155 mM^−1^ cm^−1^.

### 2.8. RNA Isolation and Gene Expression Analysis

RNAs were extracted from leaf and root tissues using RNA Plant Mini Kit (Qiagen, Cat No: 74904) according to the manufacturer’s instructions. The samples were treated with Turbo Dnase (Thermo Fisher, San Jose, CA, USA USA). RNA intactness and possible DNA contamination in the samples were checked by gel electrophoresis. The RNA amounts were determined with Qubit (Invitrogen, Waltham, MA, USA). Real time—quantitative PCR (RT-qPCR) was carried out using Rotor-Gene Q (Qiagen, Germantown, MD, USA). The expressions of the genes were quantified in 10 ng RNA samples using Luna Universal One-Step RT-qPCR Kit (NEB, Ipswich, MA, USA). The *StFH* gene forward (F: CTGCTTCTGCTTCGGAAATC) and reverse (R: AGCATCTT-GGAGAGGTGACG) primers were designed, yielding a 146 bp amplicon. The *StSec3* gene was utilized as the reference control in the study (F: GCTTGCACACGCCATATCAAT; R: TGGATTTTACCACCTTCCGCA). The *StSec3* amplicon size was 160 bp [69]. Gene expression was determined using the ΔΔ*C*_T_ method [70]. For the gene expression analysis, the *C*_T_ values of the reference gene (*StSec3*) were subtracted from the *C*_T_ of the target genes to normalize each sample. For example,

Δ*C*_T_ = *C*_T_ (*StFH*) − *C*_T_ (*StSec3*)

The value of ΔΔ*C*_T_ was obtained by comparing the Δ*C*_T_ value of stress applied plant to non-stressed plant:

ΔΔ*C*_T_ = Δ*C*_T_ (stressed plant) − Δ*C*_T_ (non-stressed plant)

The fold change for each stressed plant based on the corresponding reference (non-stressed) was calculated using the following equation:

Fold Change = 2^−(ΔΔC^_T_^)^

Average *C*_T_ values were obtained from at least six biological and four technical replicates for *StFH*.

## 3. Results and Discussion

### 3.1. Nucleotide and Protein Sequence Analyses

GC content is considered a significant feature since it affects various genomic regulations and determines the physical properties of a DNA molecule [71]. In the present study, the GC contents of potato, *Arabidopsis*, rice, and maize *FH* genes were found as 43.93%, 42.02%, 53.77%, and 51.98%, respectively. The monocots had higher GC contents than the dicots, and these variations were thought to be related to the *FH* gene’s structures. Utilizing the nucleotide sequence analysis, a total of 546 sites (excluding the ones with gaps / missing data) and 324 polymorphic (segregating) sites were identified. Among the polymorphic sites, 251 (77.5%) singleton-variable and 73 (22.5%) parsimony-informative sites were identified. The nucleotide diversity (π) was found to be 0.38. Since the queried *FH* genes were selected from both monocots and dicots, it was expected that the FH proteins from these two distinct classes naturally had distinct specificities and selectivity. Consequently, the plants from two dissimilar classes had large differences because of the balancing selection; i.e., the present variation among genes was sustained. This situation might also be originated from the low levels of both low- and high-frequency polymorphisms [72]. Overall, the *FH* genes showed no rare mutation in terms of nucleotide diversity.

**Figure 1:**
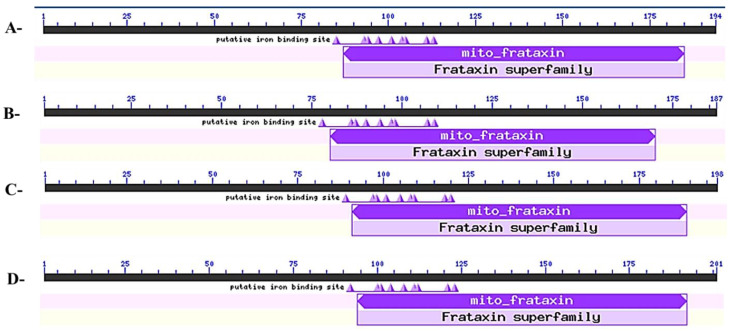
Conserved domain analysis of the FH proteins in potato (**A**), Arabidopsis (**B**), rice (**C**), and maize (**D**).

The sequence analyses of FH proteins from four plant species are presented in Table 1. Their protein lengths ranged from 187 to 201 amino acid residues, and their molecular weights were between 214.08 kDa and 220.28 kDa. All the FHs contained a conserved FH_Cyay domain (PF01491) structure (Figure 1), and their subcellular localizations were predicted as mitochondria. The *pI*s of FH proteins displayed variations, suggesting differences in the amino acid compositions. Phosphorylation events can influence the enzymatic activity of a phosphoprotein, its protein stability, and cellular localization. They may also enhance or inhibit protein binding events [73]. Phosphorylation occurs primarily at nine amino acids, including serine, threonine, and tyrosine, which are the most extensively phosphorylated. Among the different types of phosphoproteins, phosphoserines are the most prevalent, followed by phosphothreonines and phosphotyrosines. [74,75]. A total of 123 serine, threonine, or tyrosine phosphorylation sites were identified for the FH proteins, supporting their functional diversities. Five exons were identified for each *FH* gene, whereas their exon sizes were different per species, suggesting variations in the *FH* genes.

Using the identity matrix of BioEdit, potato and *Arabidopsis*, potato and rice, potato and maize, potato and soybean, potato and *S. pombe*, and potato and *E. coli* UMN026 identity values were found to be 55.3%, 51.4%, 47.7%, 56.3%, 24.2%, and 13.9%, respectively. The highest identity value was 71.6% for the maize and rice FHs, suggesting a more conserved *FH* gene structure in the monocots than in the dicots.

### 3.2. Phylogenetic Analyses

**Figure 2:**
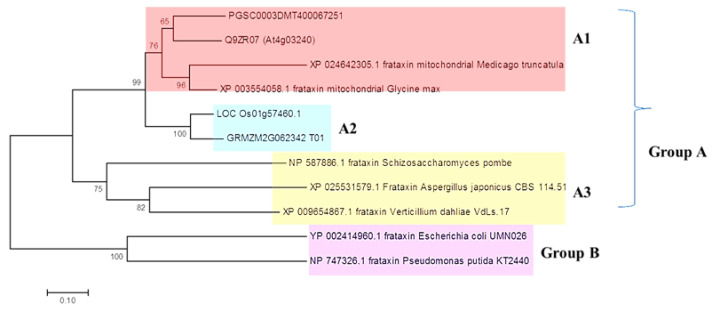
Phylogenetic relationships of monocots, dicots, fungi, and bacteria FTs using the NJ tree by MEGA7. The bootstrap values for each branch were indicated on the branch nodes.

To understand the evolutionary history of *FH* genes, phylogenetic analyses were performed, which resulted in the formation of two major groups, group A and group B (Figure 2). The main group A was divided into three subgroups: subgroup A1 of dicot plants, subgroup A2 of monocot plants, and subgroup A3 of fungi. The main group B was composed of bacterial species, and it was separated from the other main group (100%). When the phylogenetic tree was examined in general, it was observed that the separation between the monocots and dicots was very clear (100%). Eventually, it can be proposed that the *FH* genes showed a lineage-specific distribution.

### 3.3. Digital Gene Expression and Syntenic Analyses

**Figure 3:**
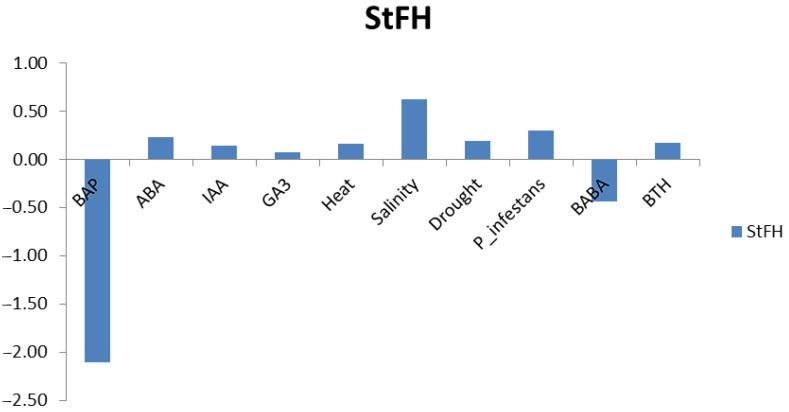
The gene expression patterns of *StFH* gene under hormone, biotic, and abiotic treatments obtained from the RNA-seq data. Expression values were indicated as log2 scale.

To provide insights into the regulation of the *StFH* gene, its expression profiles under hormone, abiotic, and biotic treatments deposited in the Potato RNA-Seq Database were examined (Figure 3). The analyses showed that the expression of the *StFH* gene increased in all the treatments except for 6-benzylaminopurine (BAP as synthetic cytokinin) and *P. infestans* elicitors DL-β-amino-*n*-butyric acid (BABA) applications. Ranging from 0.07- to 0.62-fold, *StFH* was mainly upregulated in the majority of treatments. However, the downregulations, ranging from 0.44- to 2.11-fold, were more stringent. Particularly, all of the abiotic treatments upregulated *StFH*, suggesting that it is mainly involved in abiotic stress response. Frataxin, a nuclear-encoded mitochondrial protein, has been shown to participate in Fe–S cluster biosynthesis, defend against oxidative stress, and regulate iron metabolism [13,76], supporting our findings.

### 3.4. Promotor Sequence Analyses 

First, predicted transcription factor binding sites were determined using the PlantPAN database, and the information on TF members that were particularly high in number is shown in Table 2. In particular, *WRKY*, *bZIP*, *Dof*, *bHLH*, *C2H2*, *NAC*, *Trihelix*, and *HD-ZIP* were detected as high in the number of TFs. Transcription factors (TFs) play a crucial role in regulating gene expression by binding to specific DNA sequences at the promoters of target genes. In plants, organ development is a continuous process that extends beyond the embryonic phase, and as sessile organisms, plants must adapt to a diverse array of environmental stressors. Signaling pathways controlling both development and stress response converge at the level of gene expression. Given the high proportion of TF-coding genes in plant genomes, ranging from 6–10%, transcriptional regulation may play an even more critical role in plants than in animals [77]. The obtained data from this study may support the large-scale activity of *FT* genes in cellular metabolic pathways.

To obtain more insights about *cis*-elements, stress-related *cis*-elements in the promoter regions of the *FT* genes were analyzed, as shown in Table 3. A total of seven types of stress-related cis-elements were checked, such as ABRE, SARE, G-box, W-box, P1BS, SURE, and CG-box. Notably, W-box (WRKY binding site) cis-element was found in the highest number in all four plant species. WRKY transcription factors (TFs) play a key role in regulating plant tolerance to both abiotic and biotic stresses. Cis-regulatory elements, such as the W-box (with the core sequence C/TTGACT/C), are essential for controlling gene expression in a spatiotemporal manner. Studies have shown that the tetramer sequence TGAC within the W-box is highly conserved among different plant species [78]. Considering the data obtained, it can be proposed that especially the WRKY TF family plays an active role in the regulation of FT genes in cell metabolism. 

### 3.5. Secondary and Tertiary Structure Analyses 

The biological functions, gene sequences, and protein structures proved the presence of similarities as well as variations among the *FH* gene. Protein structure and function can be good resources for neutral and adaptive mechanisms [79]. In the present study, α-helices, extended strands, beta turns, and random coils ranged from 30.35% to 43.81%, 15.98% to 21.39%, 5.67% to 6.97%, and 34.54% to 41.29%, respectively (Table 4). Therefore, the secondary structures of the proteins indicate the presence of variations among them. In addition, the 3D structures of FT proteins appear to be reliable based on the Ramachandran plot and Z-score values.

**Figure 4:**
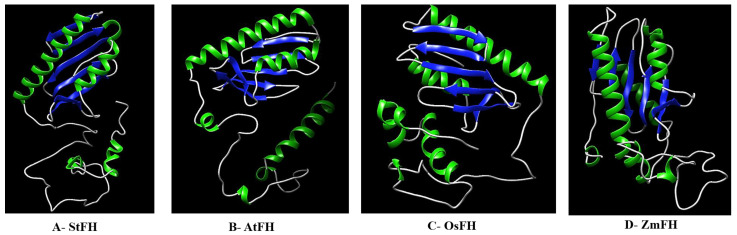
The solid ribbon representation of the predicted 3D structures of FH proteins from potato (**A**), Arabidopsis (**B**), rice (**C**), and maize (**D**).

The predicted 3D structures of FH proteins were constructed using the Phyre^2^ server (Figure 4). The comparison of 3D structures of FH proteins revealed their structural variations. Analyzed on the CLICK server, the 3D structure of StFH protein overlapped by 59.89%, 64.43%, and 64.95% with *Arabidopsis*, maize, and rice FH proteins at the tertiary level, respectively. To provide better insight into the 3D structure evolution of FH proteins, the StFH protein was compared to the FH of *E. coli*, *S. cerevisiae*, and *H. sapiens*. The FH structure overlap values were found as 83.96% between potato and *E. coli*, 86.18% between potato and *S. cerevisiae,* and 91.6% between potato and *H. sapiens* at the tertiary level. Surprisingly, the StFH protein was found to be more similar to the bacteria, yeast, and human FH proteins than the plant FH proteins. These results may be related to the high level of genetic variation of *FH* genes during evolutionary history in plant genomes. The neo-functionalization or sub-functionalization of duplicated *FH* genes may also cause those variations at the tertiary level in plants [80]. When the sequence identities were taken into consideration, the 3D structural similarities between StFH and the other FH proteins were more prevalent than their genomic sequence similarities. The sufficient conservation of FH protein can be attributed to its vital role in plants.

### 3.6. Predicted Binding Sites and Channels in FH Proteins

Proteins are smart biomolecules involved in many intra- and inter-cellular activities, such as signaling within and between cells, cellular defenses, the maintenance of the structural integrity of cells, metabolism, and catabolism. The understanding of protein–ligand interactions is important to completely understand the cellular mechanisms [81]. In the present study, the predicted binding sites of FH proteins from four plant species were identified using the InterProv75 server (Table 5). The catalytical residues in putative binding sites were distinguished with a ball-and-stick model in the 3D structures (Figure 4). Glutamic acid (E), histidine (H), and aspartic acid (D) residues were commonly found in the predicted binding sites of all FH proteins, suggesting significant contributions to the catalytic activities of plant FHs. Moreover, the residues in the catalytic sites of each protein showed variation. For instance, the 97th residue, Glu in *Arabidopsis*, was replaced by His and Asp in StFH and OsFH, respectively. On the other hand, Glu seemed to be strictly conserved in the 105^th^ positions of StFH and OsFH and 108th positions of OsFH and ZmFH (Table 5). In yeast, the conserved acidic residues of helix 1–strand 1 protein region may play roles in the iron-binding site of FH [82]. Dhe-Paganon et al. [83] reported that a cluster of 12 acidic residues was found in the first helix and the first strand of human FH at 1.8 Å resolution. In a bacterial FH ortholog (*CyaY*), several E(Glu) and D (Asp) clusters in α1 and β1 regions were identified as highly negatively charged ridges [84]. These results were in agreement with our findings.

The molecular functions of proteins are determined by their molecular structures, such as voids, channels, and pockets interacting with other molecules in their environment [66]. Identifying protein cavities, channels, and pockets that are accessible to ligands is a crucial step in predicting protein–ligand interactions [85]. In our study, varying numbers of channels were identified in the FH proteins of potato (6), maize (8), Arabidopsis (9), and rice (13), demonstrating diversity in the FH gene family (Table 6). These structural diversities may also contribute to providing functional diversities in the cellular iron metabolism in plants.

### 3.7. Physiological Analyses

**Figure 5:**
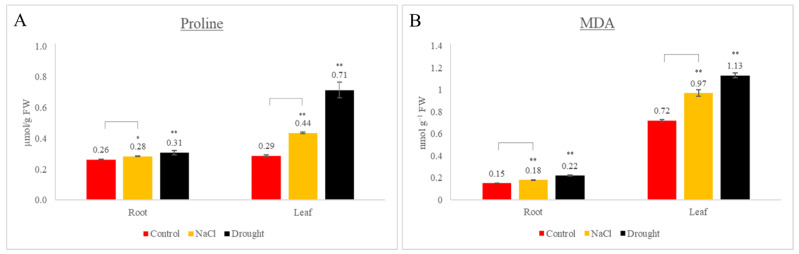
Effects of drought stress on proline (µmol/g FW) (**A**) and MDA (nmol/g FW) (**B**) concentrations of potato. Histograms represent means of six biological and four technical replicates. Error bars indicate standard error. Asterisks represent statistical significance (* *p* < 0.05 and ** *p* < 0.01; Student’s *t*-test).

To confirm the efficacy of drought and salt stresses on the plants, morphological observations, as well as physiological assays, were utilized. After 24 h of stress treatment, the effects of the stresses on the seedlings became morphologically apparent (data not shown). Afterward, the proline and MDA contents of the plants were assayed (Figure 5A,B) to evaluate if the morphological observations of the plants matched the physiological data. Proline is involved in various mechanisms to avoid the maleficent effects of abiotic stresses in plants [86]. These mechanisms vary from the adjustment of osmotic balance to the formation of antioxidants based on the stress type [87]. Proline accumulation in plants is associated with stress tolerance [88,89,90]. Research revealed that proline production was promoted and its degradation was suppressed under drought and salt stresses [91]. In the present study, it was found that proline levels in the root and leaf tissues increased under both salt and drought stresses (Figure 5A). The increases were statistically significant and confirmed the fact that proline production was induced in response to abiotic stresses. Lipid membrane peroxidation by ROS (indicated as MDA content) is a reflection of stress-induced damage at the cellular level [92]. In the present study, it was found that the MDA content was increased between 1.2- and 1.6-fold in all tissues under both stress types (Figure 5B). Although both the drought and salt stresses induced oxidative damage and lipid peroxidation in the potato cells, the effect of the drought stress was more severe than that of the salt stress, and the leaves became more affected by the stresses compared to the roots.

### 3.8. Expression Profiles of StFH under Drought and Salt Stresses

Fe, an essential element in the plant system, plays a crucial role in regulating life-sustaining processes such as respiration, photosynthesis, chloroplast development, and chlorophyll biosynthesis by participating in electron transportation. Maintaining Fe homeostasis is critical for the plant life cycle under stress conditions [93]. Frataxin, a highly conserved protein from both prokaryotes and eukaryotes, has been suggested to play multiple roles in iron metabolism, including Fe–S cluster and heme synthesis, response to oxidative damage, and oxidative phosphorylation. Plants deficient in AtFH have been found to have slower growth, increased reactive oxygen species (ROS), and oxidative stress markers [13,76]. Copper is also necessary for the assembly and function of cytochrome c oxidase, a key enzyme in the respiratory chain, in plant mitochondria [94]. Frataxins are involved in copper control in mitochondria and chloroplasts [76]. They have the ability to react with free cupric ions to form cuprous ions in vitro [95]. Chloroplasts contain over 50% of the Cu present in plants, highlighting the significance of Cu in photosynthesis [96].

**Figure 6:**
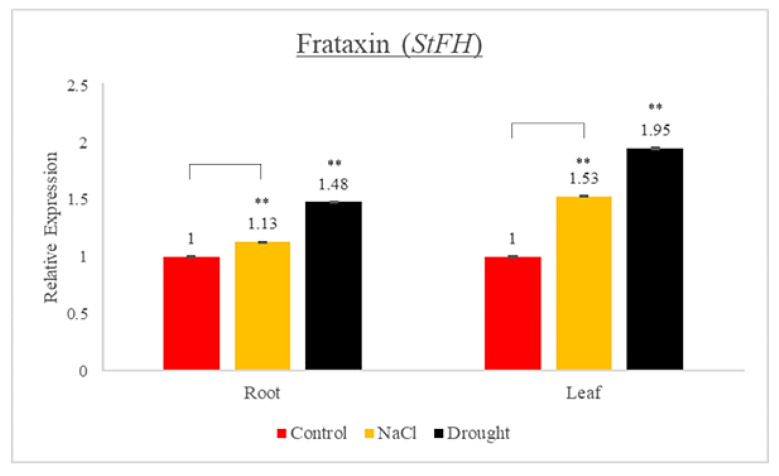
Expression profiles of StFH in potato under drought and salt (NaCl) stresses. Histograms represent means of six biological and four technical replicates. Error bars indicate standard error. Asterisks represent statistical significance (** *p* < 0.01; Student’s *t*-test).

To obtain further insights into the regulation of *StFH* in response to drought and salt stresses, the gene expressions in a potato variety were analyzed by means of RT-qPCR (Figure 6). The *StFH* was found to be 0.13- to 0.53-fold upregulated under salt stress, while it was 0.43- to 0.95-fold upregulated under drought stress. The *StFH* upregulation under both stresses was detected in both the leaves and the roots. The expressions in the leaves and under drought stress were determined to be higher than those in the roots and under salt stress, respectively. Buchensky et al. [4] reported that the expressions of the *ZmFH-1* and *ZmFH-2* genes were very limited in the roots when compared with the leaves under even regular conditions. The tissue-specific expression patterns of the *ZmFH* genes in their study correlate with our findings. Fonseca et al. [11] indicated that the *Arabidopsis* lines overexpressing the *AtFH* gene developed basal immunity and gained the ability to combat both host and non-host pathogens. Despite being limited in terms of their number, the prior studies conducted on plant FH genes, including the present study, have verified that the FH gene plays an important role in response to both biotic and abiotic stresses in plants.

## 4. Conclusions

Iron is known to be a vital nutrient for plants, and it has been shown to be involved in the structures of many biomolecules. In the present study, the *FH* gene was identified in the potato genome, and comparative bioinformatics, physiological, and expression analyses were performed. Our analyses revealed the *FH* genes to be involved in diverse biological processes, owing to the gene-duplication event stemming from evolutionary processes. We also found that the properties of the FH gene/protein varied, while the gene expression levels increased, especially under abiotic stress conditions. The level of nucleotide diversity (π) was identified as 0.38, indicating sequence variations. Despite the differences identified in their 3D protein structures, such as channel number, the active sites (particularly glutamic acid (E), histidine (H), and aspartic acid (D) residues) and domain structures (FH_Cyay domain (PF01491)) within all the *FH* genes were well conserved. The expression analyses performed in this study resulted in the upregulation of the *StFH* gene in the root and leaf tissues under both drought and salt stresses. The findings presented here are particularly important in terms of understanding the uptake mechanisms of metals such as iron and sulfur under environmental stresses in plants. They will also contribute to the understanding of the molecular role of the *FH* gene in response to abiotic stress in plants.

## Figures and Tables

**Table 1:** Sequence features of potato, *Arabidopsis*, rice, and maize FH proteins.

Phytozome ID	Species	Exon no	Protein Length (aa)	Domain Family *	Mol. wt. (kDa)	*pI*	SL	NetPhos **
PGSC0003DMT400067251	*S. tuberosum*	5	194	PF01491	217.12	7.65	M	35
At4g03240	*A. thaliana*	5	187	PF01491	214.30	5.83	M	27
LOC_Os01g57460.1	*O. sativa*	5	198	PF01491	214.08	5.10	M	29
GRMZM2G062342_T01	*Z. mays*	5	201	PF01491	220.28	5.63	M	32

* PF01491: FH_Cyay domain, SL: site of localization, M: mitochondrion, ** The NetPhos 3.1 server predicts serine, threonine, or tyrosine phosphorylation sites in eukaryotic proteins.

**Table 2:** Numbers of transcription factor binding sites (TFBSs) in the promoter regions of the FT genes obtained using PlanPAN 3.0 database.

	*WRKY*	*bZIP*	*Dof*	*bHLH*	*C2H2*	*NAC*	*Trihelix*	*HD-ZIP*
PGSC0003DMT400067251	44	39	53	7	32	38	10	43
At4g03240	93	43	35	14	32	44	12	49
LOC_Os01g57460.1	41	73	60	13	14	26	15	39
GRMZM2G062342_T01	42	26	49	11	29	54	12	6

**Table 3:** Numbers of stress-related cis-elements in the promoter regions of the FT genes obtained using PLACE database.

	ABRE	SARE	G-Box	W-Box	P1BS	SURE	CG-Box
PGSC0003DMT400067251	-	-	-	10	-	1	-
At4g03240	2	-	-	9	-	2	-
LOC_Os01g57460.1	1	-	4	20	-	2	4
GRMZM2G062342_T01	1	-	2	11	6	8	2

ABRE: ABA-responsive element, SARE: SA-responsive promoter element, G-box: environmental signal response element, W-box: WRKY binding site, P1BS: phosphate starvation-responsive element, SURE: sulfur-responsive element, CG-box: the CAMTA binding site.

**Table 4:** Analyses of secondary and tertiary structures of FT proteins in potato, *Arabidopsis*, rice, and maize.

Protein	α-Helices (%)	Extended Strand (%)	beta Turn (%)	Random Coil (%)	Ramachandran Plot (%) *	QMEAN Z-Score **
StFH	43.81	15.98	5.67	34.54	95	0.39
AtFH	33.69	18.72	6.42	41.18	97	0.39
OsFH	35.35	18.69	6.57	39.39	95	0.35
ZmFH	30.35	21.39	6.97	41.29	93	0.52

* Ramachandran plot analyses with residues in core and allowed region; ** QMEAN server used for validation of the 3D modelings of protein structures.

**Table 5:** The residue replacements of the predicted active sites of FH protein in Arabidopsis, potato, rice, and maize.

Residue Numbers	94	97	98	101	104	105	108	111
AtFH	Glu	Glu	Asp	-	-	-	-	-
StFH	Ala	His	-	Asp	Glu	Glu	-	Asp
OsFH	-	Asp	Glu	His	-	Glu	Glu	-
ZmFH	-	-	-	Glu	His	-	Glu	Glu

**Table 6:** Active-site prediction of FH protein in Arabidopsis, potato, rice, and maize.

Protein Name	Predicted Binding Sites	Function	Channel Number
StFH	85E, 93N, 94A, 97H, 101D, 104E, 105E, 111D, 113D	iron binding site	6
ZmFH	92E, 100D, 101E, 104H, 108E, 111E, 112E, 121G, 123D	iron binding site	8
AtFH	78E, 86N, 87F, 90N, 94E, 97E, 98D, 107G, 109D	iron binding site	9

## Data Availability

The datasets generated and analyzed during the current study are available from the corresponding author upon reasonable request.
